# Beyond fibrosis: the molecular roadmap from MASLD to hepatocellular carcinoma

**DOI:** 10.3389/fonc.2026.1933386

**Published:** 2026-10-05

**Authors:** Lorenzo Nevi, Annamaria Altomare, Paolo Gallo, Chiara Taffon, Teresa Musarra, Andrea Baiocchini, Simone Carotti

**Affiliations:** 1 Microscopic and Ultrastructural Anatomy Research Unit, Departmental Faculty of Medicine, Surgery and Dentistry, Università Campus Bio-Medico di Roma, Rome, Italy; 2 Gastroenterology and Endoscopy Unit, Departmental Faculty of Medicine, Surgery and Dentistry, Università Campus Bio-Medico di Roma, Rome, Italy; 3 Departmental Faculty of Science and Bio-Technology, Università Campus Bio-Medico di Roma, Rome, Italy; 4 Operative Research Unit of Clinical Medicine and Hepatology, Fondazione Policlinico Universitario Campus Bio-Medico, Rome, Italy; 5 Research Unit of Anatomical Pathology, Departmental Faculty of Medicine, Surgery and Dentistry, Università Campus Bio-Medico di Roma, Rome, Italy; 6 Anatomical Pathology Operative Research Unit, Fondazione Policlinico Universitario Campus Bio-Medico, Rome, Italy; 7 Department of Pathology, Ospedale San Camillo Forlanini, Rome, Italy; 8 Predictive Molecular Diagnostics, Fondazione Policlinico Universitario Campus Bio-Medico, Rome, Italy

**Keywords:** epigenetics, hepatocellular carcinoma, lipofagy, MASH, MASLD, metabolic reprogramming, tumour microenvironment

## Abstract

Metabolic dysfunction-associated steatotic liver disease (MASLD) has overtaken viral hepatitis as the fastest-growing cause of hepatocellular carcinoma (HCC) in most high-income countries. What makes this epidemic clinically treacherous is a feature that distinguishes MASLD-related HCC from other aetiologies: roughly half of all cases arise in livers that have never progressed to cirrhosis, slipping through the net of conventional surveillance strategies. The molecular journey from fatty liver to malignancy unfolds through at least six interlocking programmes: lipotoxic rewiring of hepatocyte metabolism, including acquired lysosomal acid lipase (LAL) dysfunction, oncogenic glucose reprogramming through the p53-PUMA-mitochondrial pyruvate carrier (MPC) axis, and a preliminary MLKL–Mfn2 axis linking metabolic stress to mitochondrial dysfunction; a chronic shift in the hepatic immune milieu toward tumour-permissive tolerance; gut microbiota dysbiosis flooding portal circulation with pro-inflammatory metabolites; progressive collapse of autophagic flux with p62/SQSTM1 accumulation and lipolysosome formation; and pervasive epigenetic rewriting spanning DNA methylation, novel histone marks, and non-coding RNA networks. We also discuss emerging biomarkers and therapeutic strategies, closing with a brief note on Doublecortin-like kinase 1 (DCLK1) as a candidate marker at the intersection of cancer stem cell identity and immune microenvironment subversion.

## 1 Introduction

Liver cancer kills more than 830,000 people each year, with HCC accounting for over three-quarters of those deaths (1). For most of the period in which its surveillance and prevention strategies were developed, that burden was concentrated in patients with established cirrhosis. Chronic HBV and HCV infection produce progressive hepatic fibrosis in a substantial proportion of carriers, and cirrhosis precedes more than 90% of virus-related HCC cases (1)—a sequential relationship that made biannual ultrasound of the cirrhotic liver a clinically coherent strategy: find the disease state that almost always comes before the cancer, surveil it, and catch tumours early. The arrival of effective antivirals and broad vaccination programmes has since reduced viral-aetiology HCC in several regions (1–4). What those successes also did, however, was leave hepatology with a surveillance framework calibrated to a carcinogenic sequence that metabolic dysfunction-associated steatotic liver disease (MASLD) does not reliably follow. MASLD now affects roughly 38% of the global adult population (5, 6) and is projected to become the leading driver of HCC within the current decade (2–4), but a substantial proportion arise before cirrhosis, including cases occurring without advanced fibrosis (2, 7). Within this broad spectrum, metabolic dysfunction-associated steatohepatitis (MASH)—defined by the coexistence of steatosis with hepatocyte ballooning and lobular inflammation—confers substantially greater oncogenic risk than simple steatosis alone: it is the sustained inflammatory insult of MASH, rather than lipid accumulation per se, that creates the hepatic microenvironment most permissive for malignant transformation (8, 9). Current guidelines have begun adjusting to this reality (3, 7), yet the cirrhosis threshold remains the principal trigger for entering surveillance—and a disease that can produce cancer before producing cirrhosis will, by design, fall through that threshold in a large fraction of affected patients.

How large that fraction is, and what the clinical consequences look like, is not self-evident from the epidemiological framing alone and requires direct evidence to appreciate. A multicentre French cohort study of 354 patients with MASLD-related HCC provides that evidence with unusual granularity: 35% of cases arose in non-cirrhotic livers, and of those patients, 72% had their tumour identified incidentally—through imaging performed for unrelated reasons—rather than through any structured surveillance programme, compared with 60% incidental detection in the cirrhotic group (10). That gap in detection pathway matters because it maps onto a gap in tumour stage at presentation: 41% of non-cirrhotic patients already harboured a lesion of 5 cm or more at diagnosis, against only 11% in patients with cirrhosis (10). These differences are consistent with, and are likely at least partly driven by, the absence of structured surveillance in many non-cirrhotic patients, although differences in tumour biology cannot be excluded. The per-patient annual HCC incidence in non-cirrhotic MASH is genuinely low in isolation—around 0.01–0.13%—but the denominator to which that rate applies runs into hundreds of millions of people worldwide, so even a small individual risk aggregates into an absolute case burden that current detection strategies were not designed to find (2, 11). The problem is unlikely to be fully addressed by simply extending existing cirrhosis-based protocols. It requires understanding, at the mechanistic level, why MASLD generates HCC through a route that does not require cirrhosis as an intermediate step.

Emphasising this fibrosis-independent route is not intended to minimise the role of fibrosis. As in other chronic liver diseases, advanced fibrosis and cirrhosis remain the dominant clinical setting in which MASLD-related HCC develops and continue to anchor current surveillance strategies. Rather, the distinctive feature of MASLD is that hepatocarcinogenesis can also arise before cirrhosis develops, creating a clinically important group of patients who are not routinely enrolled in cirrhosis-based HCC surveillance.

The four major risk loci for MASLD-HCC influence hepatocyte lipid handling and metabolic signalling, but their biology also intersects with the fibrogenic pathway. PNPLA3 rs738409 impairs triglyceride remodelling on hepatocyte lipid droplets (12), while PNPLA3 is also induced during hepatic stellate cell (HSC) activation. In primary human HSCs, PNPLA3 knockdown reduces α-smooth-muscle actin, and the I148M variant alters PPARγ and JNK/AP-1 signalling together with inflammatory and TGFβ-related responses, supporting a direct contribution to the activated myofibroblast phenotype (13). MBOAT7 is expressed in hepatocytes as well as HSCs and immune cells (14); reduced MBOAT7 activity disrupts phosphatidylinositol remodelling, promotes hepatic lipid dysregulation, and hepatocyte-specific loss can induce extracellular matrix and tissue-remodelling programmes (15, 16). TM6SF2 acts predominantly in hepatocytes, where loss of function impairs VLDL triglyceride secretion and promotes hepatic steatosis; experimental liver-specific deletion can subsequently promote fibrosis and HCC (17, 18). GCKR is likewise hepatocyte-centred and regulates the flow of glucose-derived carbon into *de novo* lipogenesis; the rs1260326 variant increases hepatic DNL, while clinical associations with fibrosis are consistent with an indirect contribution through metabolic injury rather than a demonstrated direct action on HSCs (19, 20).

The fibrotic route remains the dominant clinical context, as in other chronic liver diseases, and genetic effects on HSC activation, extracellular matrix remodelling, hepatocyte injury, and progression to advanced fibrosis can therefore contribute substantially to conventional cirrhosis-associated HCC. At the same time, the same variants can influence hepatocyte-intrinsic lipid and metabolic programmes before advanced fibrosis develops. The key distinction is therefore not that fibrosis is irrelevant, but that it is not an obligatory intermediate in every case of MASLD-associated hepatocarcinogenesis. This dual-route interpretation is reflected in the new **Figure 1** and summarised in **Table 1**.

**Figure 1:**
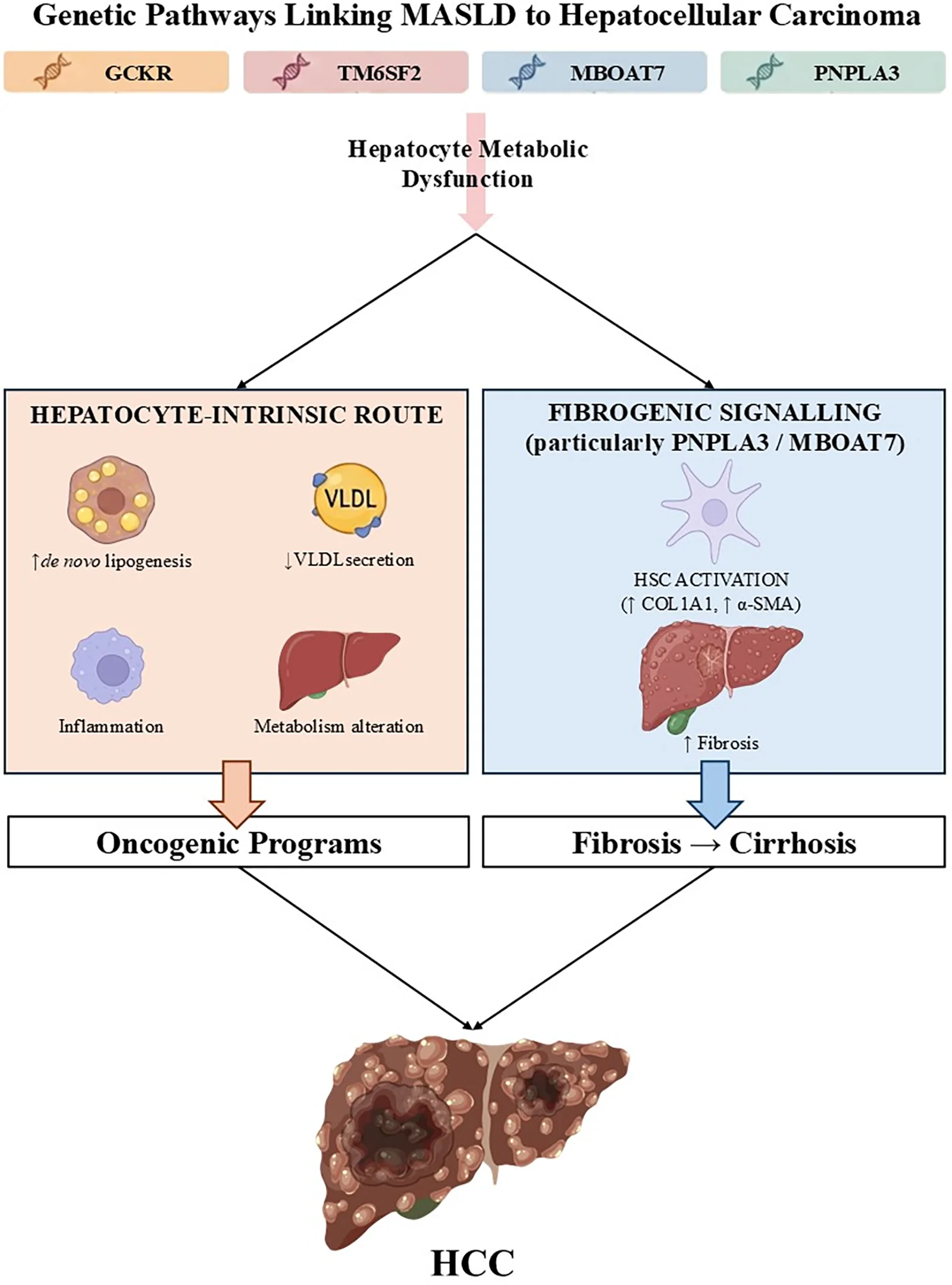
Genetic susceptibility pathways linking metabolic dysfunction, fibrosis and HCC. PNPLA3, TM6SF2, GCKR and MBOAT7 influence hepatocyte metabolic homeostasis, including lipid handling, glucose metabolism, VLDL secretion and inflammatory signalling. These alterations can contribute to hepatocyte-intrinsic oncogenic programmes, providing a mechanistic basis for HCC development before cirrhosis becomes established. In parallel, particularly through PNPLA3- and MBOAT7-associated fibrogenic mechanisms, genetic susceptibility may contribute to hepatic stellate cell activation and extracellular matrix remodelling, promoting fibrosis and cirrhosis. These pathways are not mutually exclusive and may coexist across the MASLD disease spectrum. (α-SMA: α-smooth-muscle actin; COL1A1: collagen I; VLDL: very low density lipoprotein.).

**Table 1:** Cell-type specificity and principal mechanisms of the four MASLD-HCC risk genes.

Gene	Main cellular compartment	Metabolic alteration	Link to fibrosis/HCC
PNPLA3	Hepatocyte; HSC	Impaired lipid-droplet TG hydrolysis; altered HSC lipid handling	Hepatocyte lipotoxicity and direct HSC profibrogenic effects
TM6SF2	Hepatocyte	Impaired VLDL secretion	Hepatic lipid retention → lipotoxic injury → secondary HSC activation
MBOAT7	Hepatocyte; HSC; immune cells	Altered phosphatidylinositol remodelling	Altered lipid signalling and ECM/profibrogenic responses
GCKR	Hepatocyte	Increased glycolytic/lipogenic substrate flux	Lipogenesis/lipid accumulation → hepatocyte stress → inflammatory/fibrogenic signalling

The sections that follow are organised according to a working mechanistic framework rather than a strict temporal sequence: lipid metabolic rewiring creates the lipotoxic hepatocyte environment that makes all downstream events possible; immune dysregulation of the hepatic microenvironment determines whether that environment tolerates or eliminates nascent tumour cells; gut-derived dysbiosis amplifies both signals from outside the organ; autophagic failure perpetuates lipid overload and adds a direct oncogenic input through the p62–NRF2 axis; and epigenetic reprogramming converts each of these transient metabolic perturbations into stable, heritable transcriptional alterations that persist even as the triggering stimulus fluctuates. A final section evaluates where this mechanistic understanding has translated—or is beginning to translate—into diagnostically and therapeutically actionable tools, an effort whose urgency is underscored by the surveillance gap that the preceding paragraphs have tried to document (**Figure 2**). For transparency, we clarify our approach here: literature was identified through targeted searches of PubMed/MEDLINE and Web of Science, covering publications up to July 2026, using combinations of the terms ‘MASLD’, ‘MASH’, ‘NAFLD’, ‘hepatocellular carcinoma’, and ‘non-cirrhotic’, together with mechanism-specific terms (e.g. ‘lipophagy’, ‘gut-liver axis’, ‘DNA methylation’, ‘tumour microenvironment’, ‘PNPLA3’, ‘TM6SF2’). Peer-reviewed original research, systematic reviews, and current clinical practice guidelines were prioritised; the single preprint cited in the review (the MLKL-Mfn2 mechanism) is explicitly flagged as such in the text.

**Figure 2:**
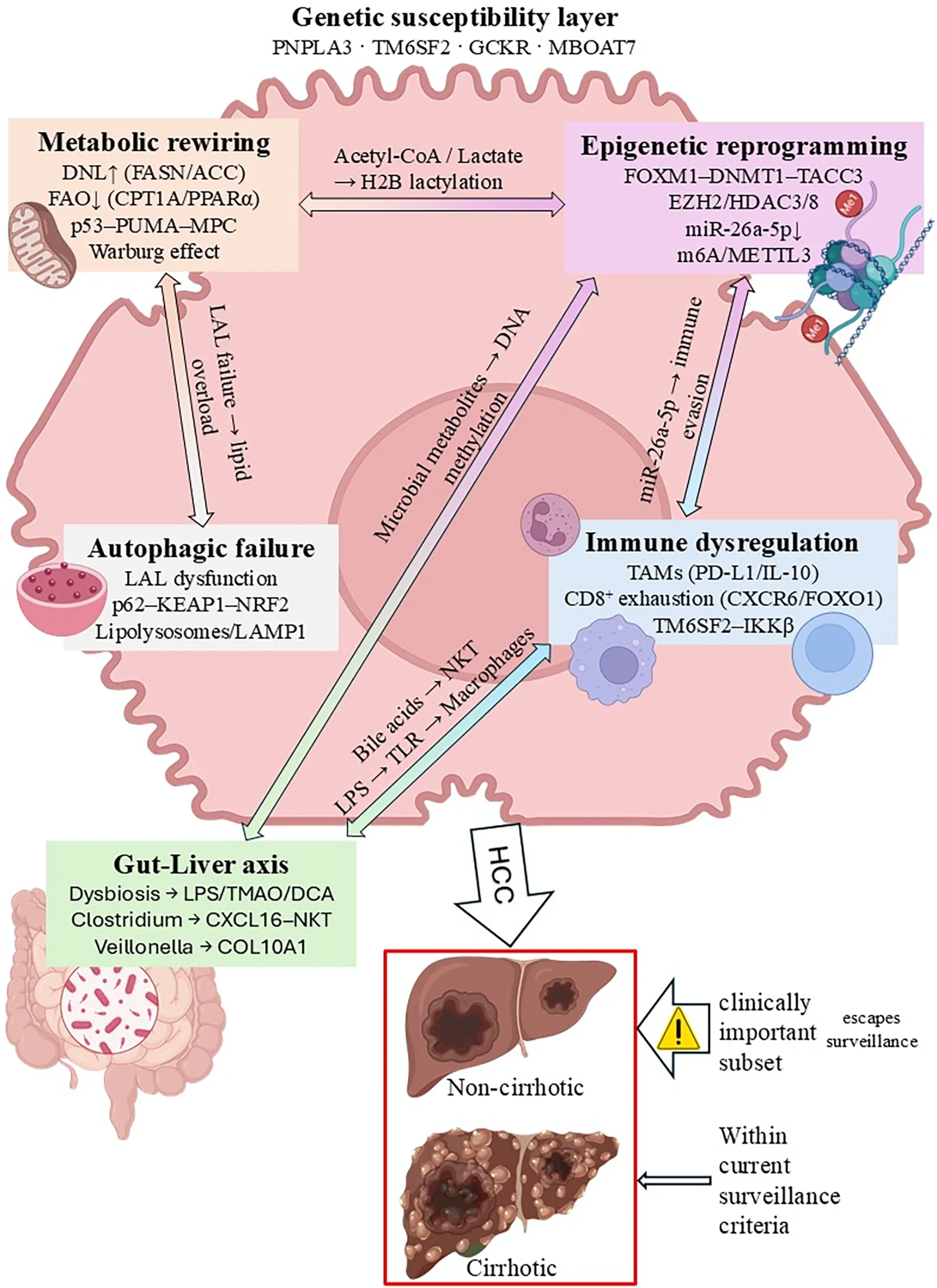
MASLD-related HCC: a tumour-permissive ecosystem beyond fibrosis. The image depicts the five interconnected pathogenic programmes contributing to HCC development in MASLD, converging on the central hepatocyte. A genetic susceptibility layer (PNPLA3, TM6SF2, GCKR, MBOAT7) modulates individual oncogenic risk. Five colour-coded compartments represent: metabolic rewiring (DNL upregulation, FAO suppression, p53-PUMA-MPC axis, Warburg effect); epigenetic reprogramming (FOXM1-DNMT1-TACC3, EZH2/HDAC3/8, miR-26a-5p loss, m6A methylation); immune dysregulation (TAM polarisation, CD8^+^ exhaustion via FOXO1-CXCR6, TM6SF2-IKKβ); autophagic failure (LAL dysfunction, p62-KEAP1-NRF2 axis, lipolysosomes); and gut-liver axis dysregulation (*Clostridium*-CXCL16-NKT, *Veillonella*-COL10A1, LPS/TMAO/DCA translocation). Bidirectional arrows denote reported or proposed mechanistic cross-talk between compartments, with the level of evidence varying across pathways. The network converges on HCC arising in a substantial proportion of non-cirrhotic livers (a clinically important subset; warning symbol), which escape conventional fibrosis-based surveillance, and in cirrhotic livers.

## 2 Metabolic dysfunction and lipotoxic reprogramming

Neoplastic cells are, above all, growth machines, and fuelling unrestrained proliferation in a lipotoxic environment requires profound metabolic reorganisation. In MASLD-HCC, this reorganisation builds on a pre-existing environment of hepatocyte lipid overload. *De novo* lipogenesis (DNL) expands dramatically through the SREBP-1c/mTOR axis, upregulating key synthetic enzymes—ACC, FASN, ACLY, and SCD1—to meet the anabolic demands of tumour growth (21–24). At the same time, CPT1A and other downstream targets of PPARα, which govern mitochondrial fatty acid oxidation, are correspondingly repressed, creating an anabolic-catabolic imbalance that favours lipid accumulation and tumour progression (21–24). Aerobic glycolysis is activated in parallel: oncogenic signals amplify glucose transporter expression and hexokinase activity, diverting pyruvate away from mitochondrial oxidation toward lactate in the classic Warburg configuration (21, 23). The resulting glycolytic intermediates supply biosynthetic building blocks—glycerol-3-phosphate, serine, ribose-5-phosphate—that sustain tumour biomass expansion (22, 24). At the genomic level, MASLD-HCC displays enrichment of mutations in ACVR2A and TP53, and tends to cluster within the proliferative S1-WNT/TGFβ transcriptomic subclass, a molecular architecture directly shaped by these metabolic pressures (25, 26).

At the organelle level, lysosomal acid lipase (LAL), which hydrolyses cholesteryl esters and triglycerides arriving at lysosomes via lipophagy, is measurably impaired in NAFLD patients independently of rare genetic LAL-deficiency (27, 28). The enzymatic shortfall traces, at least in part, to aberrant ubiquitination of the N-terminal domain of the LAL protein, which mislocalises the enzyme outside the lysosomal compartment and thereby silences the primary lysosomal fat disposal route (29). The downstream consequence is a build-up of lipotoxic intermediates that perpetuates hepatocyte injury and selects for oncogenic adaptations (27).

A more recently described and conceptually striking mechanism concerns the tumour suppressor p53, which assumes a paradoxical context-dependent oncogenic function in the metabolically dysregulated liver. During MASLD-to-HCC progression, p53 protein expression rises proportionally to the NAFLD activity score (NAS) (30). Under lipotoxic conditions, p53 transcriptionally induces PUMA, which suppresses the mitochondrial pyruvate carrier complex (MPC1/MPC2)—the gateway for pyruvate into the TCA cycle. MPC attenuation forces cells toward anaerobic glycolysis and lactate production, generating the metabolic milieu that sustains tumour growth. Both pharmacological MPC inhibition (UK-5099) and genetic MPC1 overexpression reverse these pro-tumorigenic changes in cell and animal models (30), identifying the p53-PUMA-MPC pathway as a potential therapeutic target and challenging the conventional exclusively tumour-suppressive view of p53.

A further, currently preprint-reported axis of mitochondrial disruption has been proposed to involve MLKL—a protein classically assigned to the execution of necroptotic cell death—suggested to drive HCC progression through a necroptosis-independent mechanism in the metabolically stressed liver. In this preliminary report, elevated hepatocyte MLKL is described as binding and suppressing mitofusin-2 (Mfn2), the GTPase essential for mitochondrial fusion, with proposed downstream effects on mitochondrial structural integrity and respiratory capacity, and a hypothesised contribution to cancer stem cell traits including self-renewal and cytotoxic stress resistance (31). As this evidence currently derives from a single non-peer-reviewed preprint, it should be regarded as hypothesis-generating pending independent validation. Together, LAL dysfunction and p53-PUMA-MPC rewiring—both supported by peer-reviewed evidence—converge with this preliminary mechanism to suggest a hepatocyte in which lipid disposal and, potentially, mitochondrial function are compromised—a cell primed for malignant transformation.

These metabolic derangements do not, however, operate within a cellular vacuum. Lipotoxic stress continuously generates danger signals—oxidised lipids, mitochondrial fragments, and damage-associated molecular patterns—that reshape the hepatic immune microenvironment in ways that are, as the following section shows, equally indispensable to tumour permissiveness.

## 3 The hepatic immune microenvironment

The hepatic immune microenvironment in MASLD-MASH-HCC is not organised around a single dominant cell type but rather around a layered network of myeloid cells, innate-like lymphocytes, conventional T cells, and stromal populations whose interactions collectively determine whether the liver tolerates or eliminates a nascent tumour (32–34). Hepatic macrophages are not a single entity but a shape-shifting population whose phenotype tracks—and partly drives—the stage of liver disease (35, 36). In early MASH, Kupffer cells activated by fatty acid metabolites and translocated lipopolysaccharide recruit circulating monocyte-derived macrophages; aggregates of these cells around dying lipid-laden hepatocytes—histologically visible as “crown-like structures”—sustain chronic TNF-α, IL-1β, and IL-6 secretion (35, 36). As HCC establishes itself, intratumoral macrophages undergo pro-tumorigenic reprogramming into tumour-associated macrophages (TAMs), which promote angiogenesis via VEGF and TGF-β, remodel the extracellular matrix through MMP upregulation, and impose immunosuppression through IL-10 and PD-L1 expression (35, 37). This is not a uniform population even within a single tumour: in patients with advanced HCC, infiltration by CCR2+ and CX3CR1+ macrophages tracks with non-response to immune checkpoint inhibition, whereas a CXCL10+ macrophage subset appears to predict the opposite (33, 35), a distinction that may eventually help select patients for immunotherapy rather than simply documenting why it failed.

Neutrophils add a further layer to this myeloid picture. They accumulate in the liver across multiple stages of MASH and appear to actively drive the transition from simple steatosis to steatohepatitis rather than merely responding to it (33). During tissue injury, neutrophils release extracellular traps—web-like structures of decondensed chromatin and granule proteins—that promote an immunosuppressive microenvironment through PD-L1 signalling and contribute directly to the CD8+ T-cell exhaustion discussed below (33). Pharmacologically blocking the neutrophil chemokine receptor CXCR2 interrupts this recruitment and has been shown to make NASH-HCC tumours tractable to immunotherapy in preclinical models, which is about as direct a demonstration as one could ask for that neutrophils are not bystanders in this disease (38). This is a myeloid compartment that, across multiple cell types, consistently tilts the liver toward tolerance of a developing tumour rather than its rejection.

The lymphocyte and stromal side of the ledger is equally crowded. Natural killer T (NKT) cells recognise lipid antigens presented on CD1d, and in the lipid-rich MASH liver this same recognition system appears to be hijacked: hepatocytes under metabolic stress engage NKT and CD8+ T cells through this lipid-antigen axis in a way that, rather than eliminating damaged cells, promotes steatohepatitis and tumour development through direct hepatocyte cross-talk (39). Mucosal-associated invariant T (MAIT) cells are recruited to sites of hepatic steatosis through CCR5-CCL5 signalling and, once there, secrete IL-17 and TNF-α that activate hepatic fibrotic cells; curiously, mice lacking MAIT cells develop more severe steatosis, so these cells appear to be simultaneously protective against fat accumulation and complicit in fibrosis progression (33). γδ T cells add a further innate-like layer, expanding in the MASH liver and driving steatohepatitis, hepatic injury, and disturbed glucose handling largely through IL-17A secretion, while also activating hepatic stellate cells and Kupffer cells to compound fibrotic and inflammatory damage (33). Two non-immune cell types deserve mention here too, because they actively shape rather than passively host this immune activity. Liver sinusoidal endothelial cells normally maintain an immunosuppressive, tolerogenic microenvironment; as MASH advances they undergo “capillarization”—losing fenestrae and shifting toward a pro-inflammatory phenotype marked by ICAM-1, VCAM-1, and chemokine upregulation—which both worsens hepatic lipid accumulation directly and accelerates Kupffer cell activation downstream (33). Hepatic stellate cells, better known for laying down fibrotic matrix, are themselves activated by hepatocyte-derived Hedgehog ligands and osteopontin and engage in a reciprocal loop with CD8+ T cells, which secrete IL-10 to drive stellate cell activation and proliferation in a way that worsens MASH—linking the fibrotic and immune compartments more tightly than their separate names in the literature might suggest (33). Hepatic platelets add yet another input, mediating MASH initiation and the transition to liver cancer through GPIbα signalling (40), while XCR1+ type-1 conventional dendritic cells drive a further, distinct inflammatory programme in NASH liver pathology (41).

Against this backdrop, the dysfunction and exhaustion of cytotoxic CD8+ T cells stands as a cardinal feature of progressive MASH that directly erodes anti-tumour immunosurveillance (39, 42–44). Part of this exhaustion follows a specific and somewhat unusual metabolic route: IL-15 signalling downregulates the transcription factor FOXO1 in liver-resident CD8+ T cells, which in turn upregulates CXCR6 and locks these cells into a tissue-resident state. Once resident, they respond to metabolic cues—acetate and extracellular ATP—by becoming cytotoxic toward surrounding hepatocytes in an MHC class I-independent fashion, a self-destructive programme that damages liver tissue rather than the tumour and that defines the auto-aggressive CXCR6+ CD8+ phenotype characteristic of NASH (42).

Transmembrane 6 superfamily member 2 (TM6SF2) has emerged as an unexpected brake on this broader process: by physically binding IKKβ, TM6SF2 limits NF-κB activation and curbs IL-6 output, thereby preserving CD8+ T cell effector function within the tumour (45). Hepatocyte-specific Tm6sf2 deletion in murine MASLD-HCC models accelerates tumour growth and depletes the intratumoral IFN-γ+CD8+ compartment—a phenotype that adenoviral TM6SF2 restoration reverses and that sensitises tumours to anti-PD-1 therapy (45). This positions TM6SF2 not only as a lipid metabolism regulator but as a candidate metabolic-immune regulatory node whose genetic or acquired loss contributes to the immunosuppressive phenotype of MASH-HCC.

Perhaps the most counterintuitive finding in this space is that CD8+PD-1+ T cells in MASH livers adopt an auto-aggressive phenotype co-expressing TNF, CCL2, IL-10, and granzyme B. In preclinical MASH-HCC models, prophylactic anti-PD-1 therapy paradoxically accelerated tumour formation, an effect abrogated by CD8+ T cell depletion or TNF blockade (46), suggesting that exhausted CD8+PD-1+ T cells in MASH actively promote, rather than suppress, hepatocarcinogenesis. A related but mechanistically distinct contribution comes from hepatic IgA+ cells, which arise under chronic antigenic stimulation and acquire their own immunosuppressive programme—expressing PD-L1 and secreting IL-10 in a way that directly silences cytotoxic CD8+ T cells in the tumour bed (47). Taken together, these findings argue that immunotherapy trials in MASH-HCC need patient selection criteria and endpoints distinct from those developed for viral-related HCC, where this kind of self-defeating T-cell biology has not been described (44, 46).

Many of these immune subversions—from TAM polarisation to NKT cell dysregulation and CD8+ exhaustion—are not autonomous hepatic phenomena. They are substantially orchestrated by microbial signals arriving from the intestine via the portal circuit, a regulatory axis whose dysregulation in MASLD is addressed in the section that follows.

## 4 Gut-liver axis dysregulation

The liver sits at the downstream end of the portal circuit, and for this reason it is the first organ to feel the consequences of intestinal dysbiosis (48–50). Characteristic shifts in microbiota composition in MASLD—enrichment of *Proteobacteria* and *Enterobacteriaceae* alongside depletion of short-chain fatty acid-producing taxa—compromise epithelial barrier integrity and promote systemic translocation of lipopolysaccharide, deoxycholic acid, and trimethylamine N-oxide (TMAO) into portal blood (48, 50). These microbial products activate hepatic Toll-like receptors, fuelling NF-κB- and MAPK-driven inflammatory circuits that feed forward into fibrosis and, over time, malignant transformation.

Short-chain fatty acids occupy a dual position in this axis: broadly protective during early MASLD through anti-inflammatory and barrier-sealing effects, they may paradoxically support tumour metabolic demands at advanced disease stages (48). MASH is also characterised by depletion of secondary bile acids with consequent loss of FXR-mediated hepatoprotective signalling—a further tumour-promoting dimension (51). At the microbiome-host transcriptome interface, *Veillonella* enrichment in MASLD-HCC patients specifically upregulates the oncogenes COL10A1, ENAH, and RIMS3 in peripheral blood mononuclear cells, establishing a direct causal link and identifying COL10A1 as a tissue biomarker with AUC 0.835—rising to 0.996 combined with AFP (52). The gut microbiome-liver epigenome axis constitutes a further level of control: microbial metabolites actively modulate hepatic DNA methylation and histone modification patterns, creating a microbiota-epigenetics connection that may accelerate MASLD-driven hepatocarcinogenesis (53).

Bile acids do more than signal through FXR. The G-protein-coupled receptor TGR5, expressed on Kupffer cells, enteroendocrine cells, and elsewhere in the hepatic and intestinal mucosa, responds to the same pool of secondary bile acids and tunes inflammatory tone independently of FXR (51). Depletion of secondary bile acids in MASH therefore weakens two regulatory arms at once rather than one. This bile acid pool also reaches back into the immune circuitry described in the previous section, and the mechanism here has been worked out in some detail: specific gut bacteria, most notably Clostridium species, convert primary into secondary bile acids, and the resulting shift in bile acid composition regulates CXCL16 expression on liver sinusoidal endothelial cells. Because CXCL16 is the ligand that recruits and retains CXCR6-expressing hepatic NKT cells, this microbial conversion step effectively sets the size of the liver’s NKT cell pool—and, in mouse models, depleting these Clostridium species reduces CXCL16, thins out hepatic CXCR6+ NKT cells, and allows liver tumours to grow faster, while restoring secondary bile acids reverses the effect (54). It is one of the cleaner illustrations in this field that the gut, the liver’s own lipid handling, and its resident immune compartment are not three separate problems but views of the same system from different angles.

The generation of TMAO illustrates a similarly direct line from gut microbial activity to a hepatic signal. Dietary choline and carnitine are first converted by gut bacteria into trimethylamine, which travels to the liver via the portal vein and is oxidised by flavin-containing monooxygenase 3 into TMAO (48). Because the bacterial taxa responsible for this conversion shift in a graded fashion across the MASLD-MASH-HCC spectrum—alongside the broader *Proteobacteria* and *Enterobacteriaceae* expansion already described—microbial signatures are increasingly explored not just as drivers of disease but as non-invasive biomarkers in their own right, a theme this review returns to when discussing emerging diagnostics (48). On the interventional side, non-absorbable antibiotics such as rifaximin can remodel the gut microbiome and improve liver biochemistry in MASH models, which is notable mainly because it is already in clinical use for other indications and could in principle be repurposed with comparatively little additional development risk (55). A related non-absorbable disaccharide, lactulose, offers a complementary mechanism: colonic bacteria ferment it to lactic and acetic acid, acidifying the colonic lumen, trapping ammonia as non-diffusible ammonium, and favouring saccharolytic over proteolytic flora—effects long exploited in hepatic encephalopathy that may also foster a less pro-inflammatory gut microbial and immune milieu in MASH, although direct evidence in the MASLD-HCC context specifically remains limited (56–58).

The lipid intermediates, inflammatory mediators, and microbial metabolites that gut dysbiosis delivers to the liver converge, within the hepatocyte itself, on a third axis of pathological change: the progressive failure of the autophagic machinery that normally prevents lipid burden from becoming oncogenic.

## 5 Autophagic flux impairment as an oncogenic hub

Lipophagy—the selective lysosomal degradation of lipid droplets—is the liver’s main mechanism for keeping lipid homeostasis in check (59, 60). When it fails, the consequences reach well beyond fat accumulation—they flow into the carcinogenic cascades outlined in earlier sections. That autophagic flux progressively collapses across the MASLD spectrum is now well established, grounded in converging histological, ultrastructural, and molecular evidence from experimental models and a well-characterised cohort of 59 NAFLD patients (59, 61).

The animal and cell-culture data make this point with particular clarity, because they show the same defect emerging under controlled conditions rather than only as a correlation in patient tissue. Mice fed a high-fat diet developed progressively higher hepatic p62/SQSTM1 levels and LC3-II/LC3-I ratios over 4 and then 8 months, tracking the parallel worsening of body weight, steatosis, and serum glucose, cholesterol, and transaminase levels—the longer the dietary insult, the more pronounced the autophagy block became (59). The same pattern emerged in Huh7 hepatocytes exposed to palmitic acid under high-glucose conditions: within 72 to 96 hours, p62 accumulated, the LC3-II/LC3-I ratio rose, lysosomal mass expanded, and inflammatory cytokine transcripts increased in step (59). Electron microscopy of human liver biopsies closed the loop, showing significantly more lipid droplets, lipolysosomes, and autophagosomes in NAFLD patients than in healthy controls (59). Put plainly, the autophagic machinery in a fatty liver is not idle—it is overwhelmed, and increasingly so as the disease progresses.

In human biopsies, LAMP1 immunohistochemistry identified lipid-laden lysosomes—termed lipolysosomes—whose count correlated positively with NAFLD activity score (Spearman ρ = 0.375, p < 0.005). The autophagy cargo receptor p62/SQSTM1 accumulated progressively from mild steatohepatitis to cirrhosis, forming Mallory-Denk bodies and clustering outside lysosomal structures in double-labelling experiments—indicating that the defect lies not only in autophagosome-lysosome fusion but as early as phagosome initiation (59). Parallel upregulation of lysosomal genes (CTSD, LAMP1, LAMP2, NPC1, ATP6V1B2, TFEB) and autophagy genes (ATG5, ATG7, ATG12, BECN1, RAB7A) correlated with both NAS and fibrosis stage, suggesting a compensatory but ultimately inadequate response to lysosomal overload (59, 60).

The oncogenic consequences of p62/SQSTM1 accumulation reach beyond autophagy blockade itself. When p62 sequesters the ubiquitin ligase adaptor KEAP1, it constitutively activates NRF2 and the downstream antioxidant and cell-survival transcriptional programme—conferring a growth advantage on lipotoxicity-stressed hepatocytes and directly facilitating malignant transformation (61). This p62-NRF2 axis mechanistically connects autophagic dysfunction to oncogenic permissiveness. The age dependence of lipophagy impairment—NAS and fibrosis stage were significantly worse in NAFLD patients older than 54, and p62 immunoreactivity tracked directly with patient age (59)—may partly explain why MASLD-HCC disproportionately strikes older individuals.

It helps to step back and look at what is actually breaking down. Lipophagy proceeds through a defined sequence: a portion of a lipid droplet is enclosed within a double-membrane phagophore, which matures into an autophagosome, traffics to and fuses with a lysosome, and is finally degraded by lysosomal acid hydrolases, among which cathepsin D plays a central role (60). Cathepsin D is a useful illustration of how tightly this pathway is woven into the rest of the MASLD-HCC story: its expression rises in step with NAS and fibrosis stage, and its accumulation inside overloaded lysosomes looks like both a marker of, and a contributor to, the broader lysosomal dysfunction described above (59). There is also a timing problem that complicates any simple “autophagy good, autophagy bad” framing. Early in disease, autophagy appears to support hepatocyte survival and to restrain fibrogenic signalling; its progressive failure later in MASLD removes that protective brake at precisely the point when oncogenic pressure is highest (59). This duality is part of what makes autophagy such an awkward therapeutic target—restoring flux through mTOR inhibition or AMPK activation is an appealing idea, but its benefit is likely to depend heavily on how far disease has already progressed before treatment starts (61).

The metabolic stress and oxidative burden generated by autophagic failure do not remain confined to cytoplasmic lipid handling. By depleting NAD+ and altering the flux of acetyl-CoA, lactate, and S-adenosylmethionine—the principal substrates of chromatin-modifying enzymes—they propagate their effects directly into the nucleus, initiating the epigenetic reprogramming that is the subject of the next section.

## 6 Epigenetic reprogramming

One of the more elegant insights in MASLD-HCC research over the past decade is that the relationship between metabolism and the epigenome runs in both directions (21, 62). Metabolic dysregulation alters the availability of epigenetic cofactors—acetyl-CoA, S-adenosylmethionine (SAM), lactate, and NAD+ —thereby reshaping chromatin; conversely, abnormal chromatin states lock cells into metabolic programmes that sustain disease progression. ATP-citrate lyase, which generates acetyl-CoA from mitochondrially derived citrate, links the cellular lipid surplus of MASLD directly to histone acetylation patterns (62), setting the stage for the epigenetic rewiring described below.

The best-characterised epigenetic alteration in HCC is aberrant DNA methylation: global hypomethylation coexists with focal hypermethylation of tumour suppressor gene promoters (63). In MASLD-driven disease, MASH-associated necroinflammation promotes hypomethylation-linked re-expression of oncogenes including DCAF4L2 (a member of the DCAF family of CRL4 ubiquitin ligase substrate receptors) and TUBA1B (a cytoskeletal α-tubulin isoform whose overexpression supports mitotic activity and invasiveness in HCC) (64), while RASSF1 (a Ras-association domain tumour suppressor that restrains cell-cycle progression via Hippo-pathway signalling) (65) and NOTCH3 gain silencing marks (21). The Wnt inhibitor DACT2 is epigenetically silenced in approximately 59% of HCC cases and has also been found repressed in advanced metabolic liver disease, suggesting that the same silencing event may bridge MASLD progression to frank malignancy (21). A particularly relevant MASLD-specific axis involves FOXM1 overriding DNMT1-directed methylation to force alternative splicing of TACC3, decoupling it from its physiological KAT2A-NOTCH4 regulatory role and licensing tumour-specific cell-cycle progression (66). SAM availability—controlled by the balance between MAT1A and GNMT activities—is a further metabolic-epigenetic rheostat whose dysregulation predisposes to MASLD-related carcinogenesis (67).

Chromatin regulation does not stop at DNA. EZH2-mediated H3K27 trimethylation represses Wnt pathway antagonists, amplifying β-catenin-driven transcription in MASLD-HCC (68). Class I deacetylases—particularly HDAC3 and HDAC8—contribute to insulin resistance, nuclear β-catenin accumulation, and tumour aggressiveness (69). Two more recently described histone marks are worth attention because they directly couple the metabolic state of the tumour to chromatin regulation. Lactate accumulating in the tumour microenvironment drives histone H2B lactylation at the NDRG1 locus, promoting senescence resistance (70). Separately, TGM2-mediated serotonylation of histone H3 at glutamine 5 (H3Q5ser) potentiates MYC oncogenic signalling and may prove an actionable therapeutic target (71). Both marks illustrate how novel post-translational modifications—outside the canonical acetylation and methylation framework—are emerging as important determinants of MASLD-HCC biology shaped by the metabolic microenvironment.

Non-coding RNAs add a further, more compact regulatory layer to this picture: miR-26a-5p is consistently downregulated along the MASLD-MASH-HCC axis, relieving a restraint on the β-catenin/c-Myc/EpCAM programme, and because its loss also tracks with reduced anti-tumour immune signatures it may link oncogenesis to immune evasion (72). Related regulators—miR-21-5p (73), miR-122 (74), the lncRNA NEAT1 (21), and circular RNA circMAT2B (75)—converge on the same lipid-metabolic and proliferative circuitry, and m6A methylation of transcripts such as ACLY adds an epitranscriptomic layer to the same axis (76), consistent with the broader pattern of convergent metabolism-gene alterations across chronic liver disease (25).

Crucially, many of the molecular alterations documented across the preceding sections—aberrant DNA methylation patterns, circulating miRNA signatures, specific microbial taxa, and the protein products of dysregulated metabolic pathways—are detectable in peripheral blood or tissue. They represent the mechanistic substrate from which the non-invasive diagnostic tools that the surveillance gap so urgently demands may be built, and it is to that translational effort that the review now turns.

## 7 Emerging biomarkers and therapeutic perspectives

Detecting MASLD-HCC early is harder than it should be. Conventional semiannual ultrasound suffers in obese patients from adipose acoustic shadowing, and serum AFP lacks sensitivity for small, early-stage tumours in non-cirrhotic livers. A variety of complementary strategies are being developed to fill this gap, ranging from clinical risk scores to liquid biopsy platforms, summarised in **Table 2** (77, 78). From a clinical hepatology perspective, the challenge is not to extend HCC surveillance indiscriminately to the entire non-cirrhotic MASLD population, where individual annual risk remains low, but rather to identify a biologically and clinically enriched subgroup in whom risk may justify structured surveillance (3, 7, 8, 11, 79). Current strategies still mainly rely on cirrhosis, and in selected cases advanced fibrosis, as the principal entry criterion for HCC surveillance; however, MASLD-related hepatocarcinogenesis requires a more refined risk-based approach (3, 7, 8). A pragmatic pathway should combine fibrosis assessment—through non-invasive tools such as FIB-4, liver stiffness measurement, or ELF-based algorithms (80)—with metabolic risk modifiers and genetic susceptibility (8, 81). In this framework, polygenic risk scores and similar approaches should be viewed not as immediate replacements for conventional surveillance, but as emerging tools to enrich risk prediction and select those non-cirrhotic MASLD patients most likely to benefit from closer monitoring (81). In obese patients or whenever ultrasound visualisation is suboptimal, imaging adequacy should be clearly documented and alternative strategies, including CT or MRI-based surveillance, should be considered (3, 7). The Fibrotic NASH Index (FNI) integrates routinely available AST, HDL-cholesterol, and HbA1c to flag patients with fibrotic steatohepatitis in primary care—enabling referral for closer follow-up without requiring a liver biopsy (82). At the tissue level, COL10A1 discriminates MASLD from MASLD-HCC with AUC 0.835, rising to 0.996 combined with AFP and *Veillonella* detection through microbiota-host transcriptome interaction analysis (52). Liquid biopsy platforms offer real-time molecular monitoring: circulating tumour DNA (ctDNA) detects actionable mutations in TERT, TP53, and CTNNB1 from peripheral blood; circulating tumour cells allow EMT marker profiling; and exosomal miRNA panels carry prognostic information without tissue sampling (77, 78). Multi-analyte serum algorithms—GALAD, HES v2.0, and aMAP—combine AFP, AFP-L3, and DCP with clinical variables; in a phase-3 U.S. validation study, HES v2.0 outperformed GALAD for HCC detection, including early-stage disease (83). Among specific molecular markers, Doublecortin-like kinase 1 (DCLK1) is regarded as a hypothesis-generating candidate—rather than a validated MASLD-HCC biomarker—bridging cancer stem cell identity, EMT regulation, and immune landscape composition in HCC. DCLK1 is overexpressed in HCC relative to adjacent non-tumoral tissue, bioinformatic analysis showed a correlation between DCLK1+ tumour cells and CD8+ and myeloid-derived suppressor cell infiltration patterns consistent with the MASH immune milieu (84), and was first identified in hepatobiliary malignancy as a marker of cholangiocarcinoma cancer stem cells (85). Whether DCLK1 dysregulation specifically contributes to MASLD-driven oncogenesis —rather than merely tracking it—remains an open question; we present it here explicitly as a hypothesis to guide future prospective, MASLD-specific validation, not as an established biomarker.

**Table 2:** Summary of emerging biomarkers in MASLD-associated HCC.

Category	Marker(s)	Key evidence/clinical utility	Validation status
Clinical Risk Score	Fibrotic NASH Index (FNI)	Integrates AST, HDL-C, HbA1c; identifies fibrotic NASH in primary care without biopsy (82), not validated as an HCC-specific biomarker	Validated for fibrotic MASH risk stratification; indirect relevance to HCC risk
Tissue Biomarker	COL10A1	AUC 0.835 alone, 0.996 combined with AFP and Veillonella; linked to microbiota-host oncogene network (52).	Exploratory (single-cohort discovery study)
Serum/Molecular	DCLK1, GPC3, CD276, OPN	Track immune subversion and early malignant transition; DCLK1 marks HCC stem cells and correlates with MDSC infiltration (78, 84, 85).	Exploratory/hypothesis-generating
Liquid Biopsy	ctDNA, CTCs, exosomal miRNAs	Non-invasive detection of TERT, TP53, CTNNB1 mutations; real-time disease monitoring (77, 78).	Emerging (early clinical translation)
Multi-analyte Score (AFP-based)	GALAD, HES v2.0	Integrate AFP, AFP-L3, and DCP with clinical variables; HES v2.0 outperforms GALAD in phase-3 U.S. cohort (83), though evaluated predominantly in cirrhotic populations.	Validated in phase-3, multi-cohort studies; not specifically validated for the non-cirrhotic MASLD subgroup
Risk-Prediction Score	aMAP	Relies on age, sex, albumin-bilirubin, and platelet count, without AFP-based biomarkers	Validated for general HCC risk stratification; role in non-cirrhotic MASLD not established

Several pathways described in this review translate into concrete therapeutic opportunities. Epidrugs are the most clinically advanced class: DNMT inhibitors restore silenced tumour suppressor expression and improve sorafenib sensitivity; HDAC inhibitors induce apoptosis and modulate tumour metabolism; EZH2 inhibitors restore NK cell cytotoxicity; and the dual DNMT1/G9a inhibitor CM272 shows synergistic anti-HCC activity (21). Metabolic targets include the p53-PUMA-MPC pathway—pharmacological MPC restoration reverses Warburg-type metabolism in MASLD-HCC models (30), and the DNL axis, where PPARα agonists, mTOR inhibitors, FGF21 analogues, and FASN inhibitors are progressing through preclinical and early clinical evaluation (22). The immune evasion architecture of MASH-HCC—TAMs, exhausted CD8+PD-1+ T cells, the TM6SF2 axis—argues that provides a rationale for investigating combinations of metabolic or epigenetic interventions with checkpoint inhibitors as a strategy to improve durable responses (44–46). Advances in preclinical modelling—including dietary mouse models that faithfully replicate the cardiometabolic context of MASLD, organoids, 3D-bioprinted livers, and organs-on-a-chip—are improving the translational fidelity of drug testing in MASH-HCC (86).

## 8 Conclusions and future directions

What this review makes clear is that MASLD-HCC is not a disease with a single weak point—it is a distributed network in which failure at any of several nodes (lipid catabolism, mitochondrial integrity, immune balance, autophagic flux, epigenetic fidelity) accelerates the others. This interconnection is precisely what makes it difficult to treat but also, perhaps, what makes it amenable to multi-target approaches once those nodes are properly characterised.

Three priorities stand out for the coming years. First, surveillance needs to move beyond cirrhosis as its organising principle. Non-cirrhotic MASLD-HCC will not be caught by ultrasound protocols designed for nodular, scarred livers; it requires risk stratification tools—FNI scores, liquid biopsy panels, polygenic risk scores integrating PNPLA3/TM6SF2/MBOAT7/GCKR variants—that identify high-risk patients before histological landmarks of advanced disease appear (11, 81–83). Second, the unique immune context of MASH-HCC should be factored into clinical trial design: preclinical and selected translational evidence indicates that exhausted CD8+PD-1+ T cells can behave paradoxically in this setting, suggesting that immunotherapy regimens developed for viral-related HCC may require adaptation rather than direct extrapolation—though clinical responses to checkpoint inhibition in MASLD/MASH-HCC remain heterogeneous, and this should not be read as a blanket lack of benefit from ICIs across all MASLD-HCC patients (44, 46). Third, the mechanistic connections between autophagic dysfunction, epigenetic reprogramming, and metabolic rewiring need to be studied as an integrated system rather than as isolated pathways.

Novel molecular candidates, including DCLK1 (84, 85), novel histone post-translational modifications (23, 71), specific circRNAs (75), and microbiota-derived oncogenic signals (52, 53), add depth to this picture. Bringing them from mechanistic hypothesis to validated clinical tools requires the integrated, aetiology-stratified, multi-omic longitudinal cohort studies that the field has so far lacked, supported by preclinical models that faithfully replicate the systemic metabolic context of MASLD (86). The window between silent MASLD and non-cirrhotic HCC is narrow, and the patients who fall through it have few good options once the cancer is found.

## Glossary

ACC: acetyl-CoA carboxylase
ACLY: ATP citrate lyase
AFP: alpha-fetoprotein
CPT1A: carnitine palmitoyltransferase 1A
CTSD: cathepsin D
DACT2: disheveled binding antagonist of beta-catenin 2
DCLK1: Doublecortin-like kinase 1
DNL: *de novo* lipogenesis
DNMT: DNA methyltransferase
EZH2: enhancer of zeste homolog 2
FASN: fatty acid synthase
FGF21: fibroblast growth factor 21
FNI: Fibrotic NASH Index
FXR: farnesoid X receptor
H3Q5ser: histone H3 glutamine 5 serotonylation
HCC: hepatocellular carcinoma
HDAC: histone deacetylase
HSC: hepatic stellate cell
IKKβ: IκB kinase beta
LAL: lysosomal acid lipase
LAMP1: lysosome-associated membrane glycoprotein 1
LPS: lipopolysaccharide
LSEC: liver sinusoidal endothelial cell
MAIT: mucosal-associated invariant T
MASH: metabolic dysfunction-associated steatohepatitis
MASLD: metabolic dysfunction-associated steatotic liver disease
MDSC: myeloid-derived suppressor cell
Mfn2: mitofusin-2
MLKL: mixed lineage kinase domain-like protein
MPC: mitochondrial pyruvate carrier
mTOR: mechanistic target of rapamycin
NAS: NAFLD activity score
NF-κB: nuclear factor kappa-B
NRF2: nuclear factor erythroid 2-related factor 2
OPN: osteopontin
PAMP: pathogen-associated molecular pattern
PKM2: pyruvate kinase M2
PNPLA3: patatin-like phospholipase domain-containing protein 3
PPARα: peroxisome proliferator-activated receptor alpha
PUMA: p53 upregulated modulator of apoptosis
SAM: S-adenosylmethionine
SCFA: short-chain fatty acid
SCD1: stearoyl-CoA desaturase 1
SREBP-1c: sterol regulatory element-binding protein 1c
TAM: tumour-associated macrophage
TCA: tricarboxylic acid cycle
TMAO: trimethylamine N-oxide
TM6SF2: transmembrane 6 superfamily member 2
TGR5: Takeda G protein-coupled receptor 5
FMO3: flavin-containing monooxygenase 3
NET: neutrophil extracellular trap
CCR2: C-C motif chemokine receptor 2
CX3CR1: C-X3-C motif chemokine receptor 1
CXCL10: C-X-C motif chemokine ligand 10
CXCR2: C-X-C motif chemokine receptor 2
CXCR6: C-X-C motif chemokine receptor 6
CXCL16: C-X-C motif chemokine ligand 16
MR1: MHC class I-related protein 1
NKT: natural killer T (cell)
